# *Anaplasma phagocytophilum* in the highly endangered Père David’s deer *Elaphurus davidianus*

**DOI:** 10.1186/s13071-017-2599-1

**Published:** 2018-01-08

**Authors:** Yi Yang, Zhangping Yang, Patrick Kelly, Jing Li, Yijun Ren, Chengming Wang

**Affiliations:** 1 Jiangsu Co-innovation Center for the Prevention and Control of Important Animal Infectious Diseases and Zoonoses, Yangzhou University College of Veterinary Medicine, Yangzhou, Jiangsu China; 2 Yangzhou University College of Animal Science and Technology, Yangzhou, Jiangsu China; 3 Joint International Research Laboratory of Agriculture and Agri-Product Safety of Ministry of Education of China, Yangzhou University, Yangzhou, Jiangsu China; 4 Ross University School of Veterinary Medicine, Basseterre, Saint Kitts and Nevis; 5 Dafeng Elk National Natural Reserve, Yancheng, Jiangsu China; 6 Department of Pathobiology, College of Veterinary Medicine, Auburn University, Auburn, AL USA

**Keywords:** *Anaplasma phagocytophilum*, Père David’s deer, *Elaphurus davidianus*, China

## Abstract

Eighteen of 43 (41.8%) Père David’s deer from Dafeng Elk National Natural Reserve, China, were positive for *Anaplasma phagocytophilum* based on real-time FRET-PCR and species-specific PCRs targeting the *16S rRNA* or *msp4*. To our knowledge this is the first report of *A. phagocytophilum* in this endangered animal.

## Letter to the Editor

Père David’s deer (*Elaphurus davidianus*) are now found only in captivity although they occurred widely in northeastern and east-central China until they became extinct in the wild in the late nineteenth century [1]. In the 1980s, 77 Père David’s deer were reintroduced back into China from Europe. Currently the estimated total population of Père David’s deer in the world is approximately 5000 animals, the majority living in England and China. In China 40% of the animals are concentrated in the Dafeng Elk National Natural Reserve (DENNR) which attracts over one million tourists annually.

*Anaplasma* spp. are tick-transmitted obligate intracellular Gram-negative bacteria that cause a variety of animal diseases and can also infect people [2, 3]. While control of ticks on domestic animals is time-consuming and costly, it is technically very difficult in wild animals and ticks are very common in the DENNR [4]. As *Anaplasma* infections cause considerable morbidity in animals in China [3], we tested 43 (20 males, 23 females) apparently healthy Père David’s deer for infections with *Anaplasma* spp.

DNA was extracted from whole blood samples collected from the animals with the Roche High Pure PCR Template Preparation Kit (Roche Diagnostics GmbH, Mannheim, Germany). The fluorescence resonance energy transfer (FRET) quantitative PCR targeting the *16S rRNA* gene of *Anaplasma* spp. [5] gave positive reactions for 18 deer (41.8%), including 8 females (34.8%) and 10 males (50.0%). To investigate the species of *Anaplasma* present, the positive samples were further analyzed with species-specific primers targeting the *16S rRNA* gene of *A. centrale*, *A. bovis*, *A. phagocytophilum* and *A. platys* [6, 7] as well as the *msp4* gene of *A. marginale* and *A. ovis* as described [8] (Table 1). All of the 18 positive samples were positive for *A. phagocytophilum* species-specific primers and sequencing of the PCR products with forward and reverse primers (BGI, Shanghai, China) and assembling using DNASTAR 7 revealed two different *A. phagocytophilum 16S rRNA* fragment sequences.

**Table 1:** Oligonucleotide sequences of the primers used in this study

Pathogen	Target gene	Primer		Amplicon size (bp)	References
		Primer Name	Oligonucleotide sequence (5′-3′)		
*A. centrale*	*16S rRNA*	AC1f	CTGCTTTTAATACTGCAGGACTA	426	Kawahara et al., [6]
		AC1r	ATGCAGCACCTGTGTGAGGT		
*A. bovis*	*16S rRNA*	AB1f	CTCGTAGCTTGCTATGAGAAC	551	Kawahara et al., [6]
		AB1r	TCTCCCGGACTCCAGTCTG		
*A. phagocytophilum*	*16S rRNA*	SSAP2f	GCTGAATGTGGGGATAATTTAT	641	Kawahara et al., [6]
		SSAP2r	ATGGCTGCTTCCTTTCGGTTA		
*A. marginale*	*msp4*	Amargmsp4 F	CTGAAGGGGGAGTAATGGG	344	Torina et al., [8]
		Amargmsp4 R	GGTAATAGCTGCCAGAGATTCC		
*A. ovis*	*msp4*	Aovismsp4 F	TGAAGGGAGCGGGGTCATGGG	347	Torina et al., [8]
		Aovismsp4 R	GAGTAATTGCAGCCAGGGACTCT		
*A. platys*	*16S rRNA*	Platys-F	AAGTCGAACGGATTTTTGTC	504	Inokuma et al., [7]
		Platys-R	CTTTAACTTACCGAACC		

**Fig. 1:**
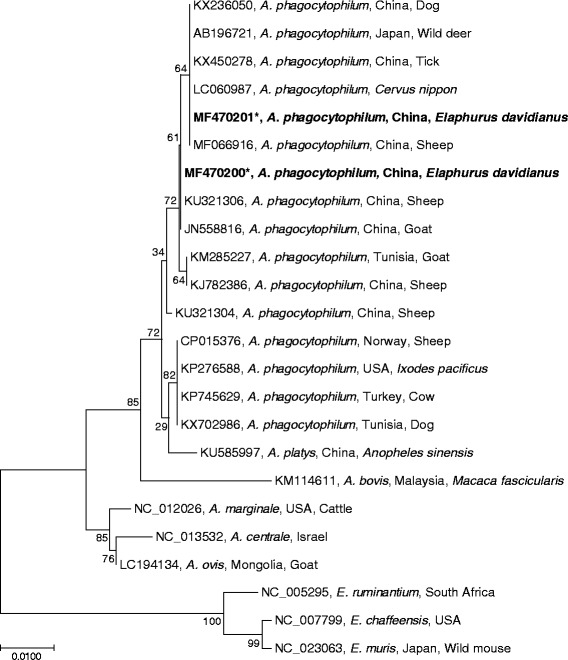
Neighbor-joining phylogenetic tree based on sequences of the *16S rRNA* gene (598–600 bp). The strains identified in our study (MF470200, MF470211) are most similar to *A. phagocytophilum*. Numbers at the branches show bootstrap support (1000 replicates). The scale-bar denotes distance

Representative sequences identified in our study (MF470200 and M470201) were aligned using CLUSTAL W in MEGA 7 with those of 14 *A. phagocytophilum* and sequences for *A. platys*, *A. bovis*, *A. marginale*, *A. centrale*, *A. ovis*, *Ehrlichia ruminantium*, *Ehrlichia chaffeensis* and *Ehrlichia muris* retrieved from GenBank (Fig. 1). Phylogenetic analysis demonstrated that the *Anaplasma* isolates studied were all closely related to *A. phagocytophilum* based on the *16S rRNA* gene sequences (99.2–100% similarity, 598–600 nucleotides). Compared with those of other *Anaplasma* spp., the similarity was 99.0% with *A. platys* (6 mismatches/599 nucleotides), 96.7% with *A. bovis* (20 mismatches/599–600 nucleotides), 97.2% with *A. marginale* (16 or 17 mismatches/599 nucleotides), 96.7% with *A. centrale* (19 or 20 mismatches/599 nucleotides), and 97.4% with *A. ovis* (15 or 16 mismatches/599 nucleotides), respectively.

To our knowledge, this is the first report of *A. phagocytophilum* in Père David’s deer, a nationally protected species in China. There is little data on the pathogenic effects of *A. phagocytophilum* in deer although a wide variety are susceptible and some species can have prolonged infections and are most likely reservoir hosts [9]. Brown rat (*Rattus norvegicus*), black-striped field mouse (*Apodemus agrarius*), common pheasant (*Phasianus colchicus*) and Siberian thrush (*Zoothera sibirica*) that have  been reported as hosts for *A. phagocytophilum* [10–12] are found to reside in DENNR. In domestic ruminants in Europe, however, infections are associated with fever and anorexia, abortion storms, occasional deaths, decreased milk production and immunosuppression [9]. Further studies are required to determine if the *A. phagocytophilum* infections we observed in the Père David’s deer might be detrimental to the survival of the species.

*Haemaphysalis longicornis* is prevalent in the DENNR (summer: 89.5 ± 17.1 ticks/10 m^2^, winter: 1.47 ± 0.35 ticks/10 m^2^) and is the only reported tick species found on the Père David’s deer in the reserve [4]. Although *Ixodes persulcatus* is usually associated with *A. phagocytophilum* in Asia, this tick is distributed in the north of China and, along with other ticks reported to be infected - *Dermacentor silvarum*, *Ixodes ovatus*, *Ixodes niponensis*, *Haemaphsalis megaspinosa* and *Haemaphyslais douglasii* - has not been reported in the DENNR reserve. *Haemaphysalis longicornis* has been found to be infected with *A. phagocytophilum* and *A. bovis* in China and other regions [13–16] and appears to be the most likely source of the infections identified in our study.

Humans can also be infected with *A. phagocytophilum* resulting in human granulocytic anaplasmosis which might be asymptomatic or a mild febrile illness with headache, malaise, and myalgia [17]. Uncommonly it might cause severe disease with multiple organ failure and death although mortality rates might be significantly higher in China (27%) [18]. Our finding of infected deer and the reported high prevalence of *H. longicornis* which is known to feed on humans should alert health professionals to the possibility of *A. phagocytophilum* infections in patients with a history of visiting the DENNR.

To the best of our knowledge, this is the first report of *A. phagocytophilum* in Père David’s deer. Further studies are needed to determine the effects on these infections on this endangered species and the role they might play in the epidemiology of human infections.

## Abbreviations

DENNR: Dafeng Elk National Natural Reserve
FRET: fluorescence resonance energy transfer
